# Hybrid Devulcanized/Vulcanized Crumb Rubber Strategy for High-Performance Asphalt with over 40% Recycled Tire Rubber Content

**DOI:** 10.3390/polym17222987

**Published:** 2025-11-10

**Authors:** Zhengkun Wang, Ruihuan Wang, Heng Zhang, Bo Zhang, Yinghua Fan, Wenwen Yu, Qiang Zheng, Fengbo Zhu

**Affiliations:** 1 College of Materials Science & Engineering, Taiyuan University of Technology, Taiyuan 030024, China; 2 Shanxi Transportation Technology Research and Development Co., Ltd., Taiyuan 030032, China; 3 The Key Laboratory of Road and Traffic Engineering, Ministry of Education, Tongji University, Shanghai 201804, China; 4 Department of Polymer Science & Engineering, Zhejiang University, Hangzhou 310027, China

**Keywords:** modified asphalt, high crumb rubber content, vulcanized/devulcanized crumb rubber hybridization

## Abstract

Utilizing waste tire crumb rubber (CR) in asphalt modification is a promising method to enhance pavement performance while addressing the issue of waste tire disposal. Elevating CR content without compromising the pavement performance of asphalt is crucial for its practical and sustainable applications. However, conventional crumb-rubber-modified asphalt (CRMA) exhibits weakened physical and pavement properties when the CR content exceeds 25 wt%. Here, we propose a hybridization strategy combining CR and devulcanized CR (DCR) to produce high-performance modified asphalt with a total rubber content of up to 43 wt%. Modified asphalt containing 30 wt% CR and 13 wt% DCR (30CR-13DCRMA) demonstrates remarkable physical properties, with a softening point of 78.4 °C and a ductility of 15.33 cm. Rheology tests further reveal its superior rutting resistance (G*/sin δ), fatigue tolerance (G*·sin δ), and overall pavement performance compared to neat CR- or DCR-modified asphalt. Through rheological analysis, sol fraction measurement, gel permeation chromatography (GPC), and atomic force microscope (AFM) tests, it is revealed that the synergistic effect of CR and DCR can enhance the absorption capabilities of rubber particles, promoting their full swelling and resulting in a biphasic hard/soft microstructure within the asphalt matrix. This structural reorganization contributes to the outstanding comprehensive properties of this modified asphalt. This work establishes a hybrid-rubber asphalt system with high CR incorporation and well-balanced performance, offering a viable pathway toward sustainable pavement engineering.

## 1. Introduction

The global demand for petroleum asphalt continues to grow at approximately 10% annually, driven by the construction and maintenance of transportation infrastructure. However, as a non-renewable resource, petroleum asphalt faces long-term supply challenges [1]. Additionally, the inherent limitations of asphalt, such as susceptibility to high-temperature rutting, low-temperature cracking, and fatigue cracking, significantly compromise pavement durability and service life [2]. These issues underscore the need for sustainable strategies to enhance their performance while reducing reliance on petroleum asphalt [3,4].

Currently, the most widely used asphalt modifiers include styrene-butadiene-styrene (SBS) block copolymers and recycled tire-derived crumb rubber (CR). SBS, a thermoplastic elastomer with a biphasic structure, can offer superior elasticity and mechanical strength to asphalt [5]. However, its high cost and low incorporation level (typically below 5 wt%) limit its potential for large-scale replacement of petroleum asphalt [6]. In contrast, CR, produced by grinding waste tires, offers a cost reduction of 70–80% compared to SBS and can be incorporated into asphalt at 10–20 wt%, simultaneously improving asphalt performance and addressing environmental challenges posed by waste tires [7].

The vulcanized cross-linked network structure of CR contributes to excellent mechanical strength, wear resistance, and elasticity, which can significantly enhance the high/low-temperature performance and fatigue crack resistance of crumb-rubber-modified asphalt (CRMA) [8,9,10]. Moreover, sulfur, carbon black, and inorganic oxides released from CR during blending can reduce the high-temperature susceptibility while enhancing aging resistance of asphalt [11]. Rheological studies reveal that CRMA exhibits reduced phase angles, increased shear modulus, higher rutting resistance, and substantially lower non-recoverable strain in multiple stress creep recovery (MSCR) tests compared to base asphalt [12]. These advantages highlight the promising application of CRMA in road engineering.

Despite the potential economic and environmental benefits of CR [13,14], the road performance and storage stability of CRMA deteriorate drastically when CR content exceeds 25 wt% [15,16]. This limitation stems from three main factors: (1) As its content exceeds over 25 wt%, the added CR particles cannot reach their fully swollen states by fully absorbing light components from base asphalt, which could weaken the elastic core functions of swollen rubber particles. (2) The weak interfacial adhesion between excessive CR and asphalt leads to inefficient stress transfer capability [17]. (3) When CR content exceeds over 25 wt%, the poor compatibility between CR and asphalt often results in phase separation during prolonged storage, further impairing its performance [18,19].

To enhance CR–asphalt compatibility and increase CR incorporation levels, various modification methods have been proposed, including devulcanization, mechanochemical treatment, polymer coating, grafting/interpenetrating polymer networks, and organic–inorganic hybridization [20,21,22,23,24,25]. Among these, devulcanization is the most common approach, which breaks the cross-linked network (e.g., breaking C-C, C-S, and S-S bonds) to improve CR dispersion and compatibility with asphalt [26]. However, devulcanized crumb rubber (DCR) suffers from reduced high-temperature performance due to network degradation [27,28], and its production is energy-intensive and generates significant wastewater and emissions, posing environmental concerns [29,30].

To address these challenges, this study proposes a hybrid strategy to achieve high-performance asphalt with over 40 wt% of CR by blending DCR with untreated CR at an optimized ratio. This approach enables the balance of swelling and interfacial compatibility while minimizing DCR usage, thereby reducing energy consumption and environmental impacts. The fundamental properties and rheological behavior of the modified asphalt were systematically investigated. Furthermore, atomic force microscopy (AFM) and gel permeation chromatography (GPC) were employed to elucidate the interaction mechanisms between CR and asphalt.

## 2. Materials and Method

### 2.1. Materials

Base asphalt (90^#^ penetration grade) was purchased from Sinopec Group (Beijing, China). Crumb rubber (CR) was supplied by Haiyi Co., Ltd. (Shenzhen, China), which was produced at 1.2 tons/h through sectioning of rubber blocks followed by pulverization into approximately 40-mesh particles. Devulcanized crumb rubber (DCR) was purchased from Jiangsu Zhonghong Environmental Technology Co., Ltd. (Jiangyin, China). Corresponding specifications are detailed in Table 1 and Table 2.

### 2.2. Preparation of Rubber-Modified Asphalt

**Figure 1:**
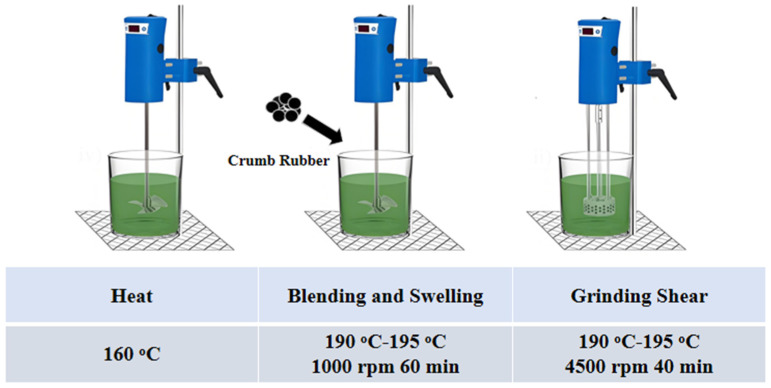
Production of high-content rubber-powder-modified asphalt.

The asphalt was pre-melted at 100 °C and then transferred into a metal container. Rubber particles at certain CR/DCR ratios (Table 3) were further blended into asphalt at a higher temperature. It was found that the modified asphalt could not be processed as the temperature was lower than 190 °C, while a higher temperature could lead to severe degradation of the materials (Table S1). In Figure 1, the processing temperature was set between 190 and 195 °C. The modified asphalt was stirred at 1000 rpm for 1 h and then sheared using a high-speed shear emulsifier at 4500 rpm for 40 min to obtain modified asphalt samples.

### 2.3. Characterization

(1) **Physical properties**

The main physical properties of CRMA, such as penetration (25 °C), softening point, ductility (5 °C), viscosity (180 °C), elastic recovery (25 °C), and storage stability at 163 °C, were tested according to the JTG E20-2011 standards for highway engineering asphalt and asphalt mixtures [31].

(2) **Rheological test**

Temperature sweep tests were conducted from 52 °C to 82 °C using a rotational rheometer (MCR 102e, Anton Paar, Graz, Austria), with the temperature incrementally increased following AASHTO T315 standards [32] in 6 °C intervals. Samples were positioned between 25 mm diameter parallel plates with a 1 mm gap. A sinusoidal oscillatory load was applied to the upper plate at a fixed frequency of 10 ± 0.1 rad/s. The complex shear modulus (G*), phase angle (*δ*), and rutting factor (G*/sin δ) were measured to assess the high-temperature performance of the modified asphalt.

The rheological properties of modified asphalt were also characterized according to AASHTO T315 standards using a Dynamic Shear Rheometer (ARES-G2, TA Instruments, New Castle, DE, USA) equipped with 8 mm parallel plates and a 2 mm gap. The linear viscoelastic region (LVER) was first evaluated through strain sweeps at 10 °C and 10 rad/s, identifying 10% strain as the upper LVER limit. Subsequent frequency sweeps (0.1–100 rad/s) were conducted across a temperature range of −10 °C to 10 °C. Intermediate-to-low-temperature performance was evaluated using the fatigue parameter (G*·*sin δ*) at the reference temperature of 10 °C, derived through time–temperature superposition (TTS) analysis within the instrument’s software [33,34].

(3) **Phase separation of CRMA and calculation of particle effect (*PE*)/interaction effect (*IE*)**

The particle effect (*PE*) and interaction effect (*IE*) were employed to quantify particulate and interfacial contributions to high-temperature performance of the modified asphalt.

Building on previous methodologies, swollen rubber particles were isolated from rubber-modified asphalt via filtration to extract the residual asphalt matrix for subsequent analysis. PE and IE values were calculated as follows [35]:

(1) $$PE=\frac{P_{AR}-P_{LP}}{P_{BA}}$$

(2) $$IE=\frac{P_{LP}-P_{BA}}{P_{BA}}$$

where *P_AR_*, *P_LP_*, and *P_BA_* represent the specific rheological properties of CRMA, liquid phase, and base asphalt, respectively. The rutting factor was employed in this study [36].

(4) **Multiple stress creep recovery (MSCR) test**

The MSCR test was conducted using a rotational rheometer (MCR 102e, Anton Paar, Austria) equipped with 25 mm parallel plates and a 1 mm gap. The tests were performed according to AASHTO T 350-19 standards at 64 °C [37]. The recovery percentage (R) and non-recoverable creep compliance ($J_{nr}$) were calculated for each stress level according to the following:

(3) $$R=\frac{\varepsilon_{p}-\varepsilon_{u}}{\varepsilon_{p}}\times 100\%$$

(4) $$J_{nr}=\frac{\varepsilon_{u}}{\sigma }$$

where $\varepsilon_{p}$ is the peak strain of each cycle; $\varepsilon_{u}$ is the unrecovered strain; and σ is the stress level, 0.1 kPa and 3.2 kPa.

(5) **Gel permeation chromatography**

To perform the GPC test, the modified asphalt was dissolved in tetrahydrofuran (THF) to prepare solutions (50 mg/mL) which served as the mobile phase and were equilibrated at room temperature for 12 h to ensure complete dissolution. The dissolved solutions were injected into a gel permeation chromatograph (GPC, Waters 1515, Milford, MA, USA) and analyzed over a period of 25 min.

(6) **Atomic force microscopy (AFM)**

Atomic force microscopy (AFM, MFP-3D Origin, Oxford Instruments, Oxford, UK) was used to observe the microscopic morphology of modified asphalt at room temperature. To prepare the samples, the modified asphalt was deposited on a glass slide and melted on a 100 °C hot stage, after which the samples were smoothed and cooled. Scanning was performed over 30 µm × 30 µm areas using an AC160TS-R3 probe (OLYMPUS, Shinjuku, Japan), with surface smoothness maintained to prevent tip damage.

(7) **Solubility test**

Undissolved CR particles within the asphalt matrix significantly influence CRMA performance [38]. To isolate these particles from CRMB, filtration was performed using a laboratory-grade sieve. Specifically, 10 ± 0.1 g of CRMB was dissolved in 150 mL of dichloromethane and maintained at room temperature for 1.5 h. The solution was then filtered through a 45 μm mesh sieve [39]. The residual CR particles on the sieve were rinsed with solvent and dried at 75 °C until constant weight was achieved. The solubility, defined as the percentage of dissolved CR mass, relative to the total incorporated CR mass, is expressed as follows [40]:

(5) $$S=1-\frac{1+\omega }{\omega \cdot m_{0}}\cdot m_{1}$$

where *S* is the solubility (%), ω is the mass fraction of the CR particles within the bituminous matrix (%), $m_{0}\;$is the mass of the CRMA binder (g), and $m_{1}$ is the mass of the residual CR after filtration (g).

## 3. Results and Discussion

### 3.1. Effect of High-Content Hybrid Rubber on Fundamental Properties of Asphalt

**Figure 2:**
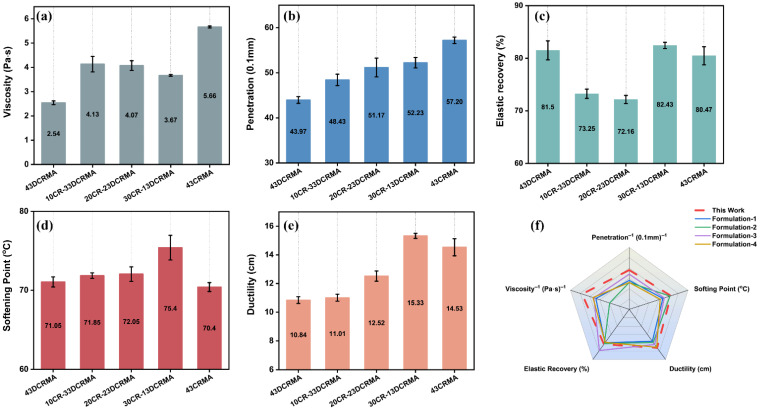
Fundamental physical properties of rubber-modified asphalt with varied CR to DCR ratios; the total rubber content is fixed at 43%. (**a**) Viscosity (180 °C); (**b**) penetration (25 °C); (**c**) elastic recovery (25 °C); (**d**) softening point; (**e**) ductility (5 °C); (**f**) comparative diagram of fundamental properties.

Figure 2 illustrates the influence of varying CR/DCR ratio on the viscosity and fundamental physical properties of modified asphalt; the total rubber content is fixed at 43 wt% (Figure S1). As shown in Figure 2a, the viscosity of hybrid-rubber-modified asphalt falls between 43DCRMA and 43CRMA. Previous research associates viscosity with the extent of swelling degradation of rubber and its compatibility with asphalt [41]. Typically, DCR undergoes severe degradation during the preparation process, which compromises its inherent elasticity and toughening effect on asphalt. Conversely, CR exhibits poor compatibility with base asphalt at high loading contents, resulting in phase separation and higher viscosity, thereby impairing its processibility [42]. The intermediate viscosity observed in the hybrid CR/DCR-modified asphalt indicates that rubber particles underwent moderate degradation, which may have helped preserve their inherent elasticity and improved the physical properties of modified asphalt.

As shown in Figure 2b–e, the hybrid strategy effectively improved the physical properties of rubber-modified asphalt, even though the loading content was as high as 43%. At the CR/DCR ratio of 30/13, the modified asphalt demonstrated exceptional overall performance, with a penetration of 5.22 mm, elastic recovery of 81.81%, softening point of 75.4 °C, and ductility of 15.33 cm, respectively. This result indicates that although prolonged high-temperature blending at around 190 °C may lead to the emission of oils and small-molecule gases, posing potential environmental risks [43], the hybridization of vulcanized/devulcanized rubbers can effectively preserve the three-dimensional network structure of the rubber, thereby minimizing the leakage of harmful substances. This approach simultaneously reduces asphalt consumption, enhances the pavement performance of modified asphalt, and extends its service life in road construction.

We further compared the processibility and physical properties of the prepared rubber-modified asphalt with existing studies on high-content rubber-modified asphalt. Here, the reciprocals of viscosity and penetration were employed as indicators to evaluate workability and high-temperature resistance, respectively. Compared with the hybrid devulcanized/vulcanized crumb rubber strategy in this study, earlier formulations for high-rubber-content asphalt improved performance mainly through rubber activation or by adding small-molecule additives. As shown in Figure 2f, the hybrid strategy yields high-content rubber-modified asphalt with competitive overall performance [44,45].

**Figure 3:**
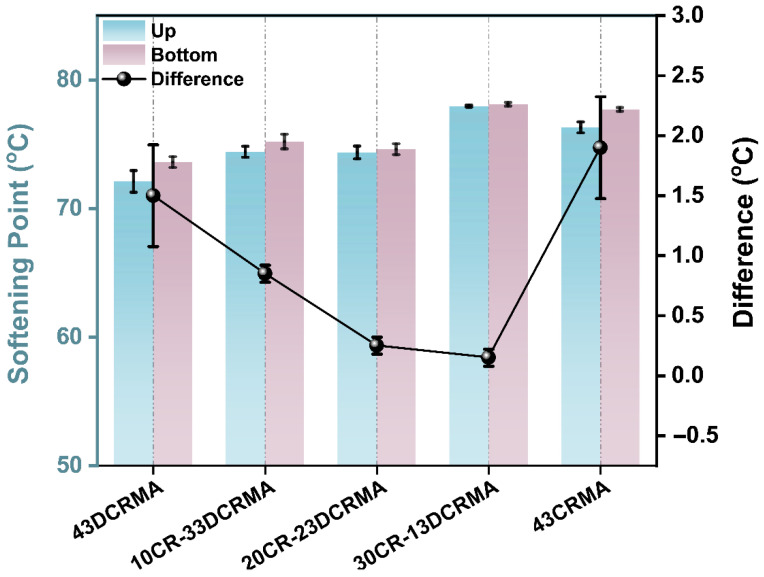
Storage stability at 163 °C of rubber-modified asphalt with varied CR ratios.

Figure 3 presents the softening point difference between the top and bottom sections of CR/DCR-modified asphalt and serves as an indicator of its storage stability. Both 43CRMA and 43DCRMA exhibit remarkable softening point differences, indicating their poor storage stability. This is because neat CR exhibits poor compatibility with base asphalt, which may cause pronounced phase separation and segregation. Conversely, the severe degradation of rubber particles in 43DCRMA results in sedimentation, which also produces large softening point difference [46]. Notably, the hybrid CR-DCR rubber strategy effectively improves storage stability of the modified asphalt. At an optimal CR/DCR ratio (30:13), the reduced relative content of vulcanized rubber enables more complete absorption of the light components from the base asphalt. This promotes improved swelling of the rubber particles, effectively mitigating particle settling and enhance the storage stability of the modified asphalt [47,48].

### 3.2. Rheological Properties and Rutting Resistance

**Figure 4:**
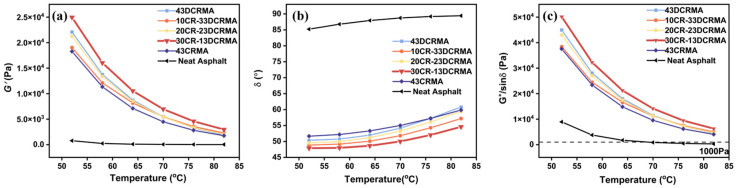
Temperature sweep of rubber-modified asphalt with varied ratios: (**a**) energy storage modulus; (**b**) phase angle; (**c**) rutting resistance.

Figure 4 presents the evolution of elastic modulus (*G′*), phase angle (*δ*), and rutting resistance (G*/sin *δ*) under temperature sweep tests for modified asphalt with different CR/DCR ratios. Compared to neat CR- and DCR-modified asphalt, the hybrid strategy significantly enhances elasticity, as indicated by a notable increase in *G′* and a decrease in *δ* of the 30CR-13DCR-modified asphalt. The highest rutting resistance was observed in 30CR-13DCR-modified asphalt, which also demonstrates the effectiveness of the hybrid strategy in enhancing high-temperature deformation resistance. This improvement can be attributed to the cross-linked rubber particles, which absorb the light components within swelling asphalt, thus acting as elastic fillers to give better elasticity to the modified asphalt. Meanwhile, as the CR ratio increases, the swollen rubber particles have reduced interparticle distances, forming an interconnected three-dimensional network. This structure imparts the asphalt matrix with improved deformation recovery and enhances rutting resistance. Nevertheless, as the amount of CR further increases (43CRMA), the incorporated rubber particles cannot fully absorb those light components from the base asphalt, which affects their elasticity, causing a weakening in the performance of modified asphalt [49].

### 3.3. 10 °C Frequency Scan Analysis

**Figure 5:**
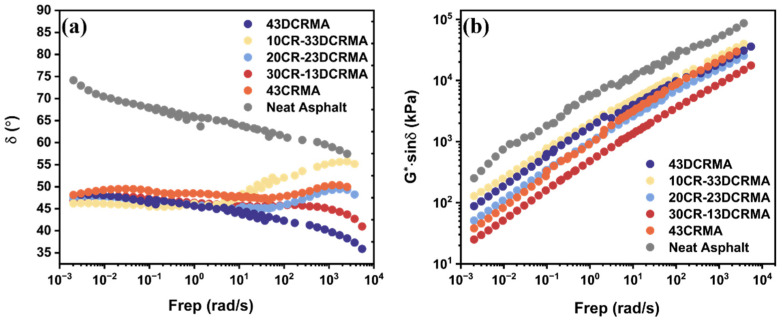
Frequency scan of rubber-modified asphalt with varied ratios at 10 °C: (**a**) phase angle; (**b**) fatigue factor.

Figure 5a shows the phase angle master curve obtained using the time–temperature superposition (TTS) principle at a reference temperature of 10 °C. The addition of crumb rubber enhances the elasticity of neat asphalt, reducing its phase angle. When DCR is used, the phase angle increases markedly at lower frequencies because the devulcanization breaks the cross-linked structure of rubber particles, enhancing their molecular mobility and temperature sensitivity [50]. As the CR ratio increases, the phase angle curve gradually flattens, indicating the hybrid strategy enhances elastic performance over a wide frequency range. Figure 5b shows the fatigue factor (G*⋅sin *δ*) of rubber-modified asphalt with varying CR/DCR ratios at 10 °C. Since a lower G*⋅sin *δ* value indicates better resistance to fatigue cracking, here, 5000 kPa is used as a threshold. It is revealed that 43DCRMA exhibits a high fatigue factor, suggesting that it is brittle at low temperatures [51]. However, blending an appropriate ratio of cross-linked CR (30CR-13DCRMA) results in the lowest fatigue factor. This improvement can be attributed to the formation of a fatigue-resistant network within the asphalt matrix, thus enhancing its fatigue resistance [52].

### 3.4. Multiple Stress Creep Recovery (MSCR) Analysis

**Figure 6:**
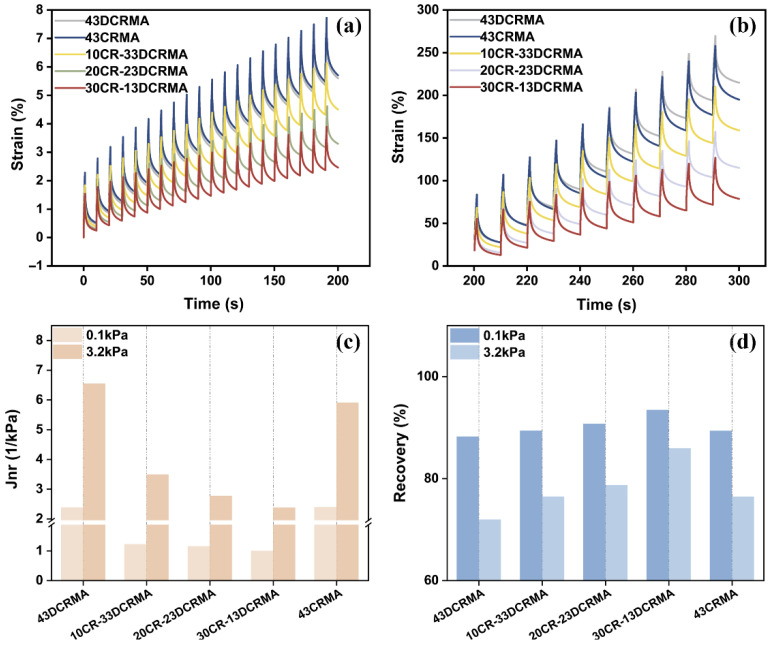
MSCR of rubberized asphalt with varied CR ratio at 64 °C: (**a**) accumulated strain curves at 0.1 kPa; (**b**) accumulated strain curves at 3.2 kPa; (**c**) non-recoverable creep compliance; (**d**) recovery percentage.

Multiple stress creep recovery (MSCR) tests were conducted at 64 °C to evaluate the high-temperature performance of the modified asphalt under simulated traffic conditions. Figure 6a plots the cumulative strain curves during loading (0–200 s) for the first to twentieth cycles at 0.1 kPa, simulating light traffic conditions. Figure 6b presents the cumulative strain under 3.2 kPa, simulating heavy traffic conditions over 10 loading cycles (200–300 s). At identical stress levels, increasing the CR ratio in the high-content rubber-modified asphalt significantly reduces non-recoverable cumulative strain, demonstrating improved high-temperature rutting resistance. This observation is consistent with previous findings from temperature sweep tests.

Non-recoverable creep compliance (Jnr) reflects the resistance to permanent deformation under cyclic loading conditions, while recovery percentage quantifies elastic performance. As shown in Figure 6c,d, both properties improve progressively with higher CR ratios, reaching an optimum in the 30CR-13DCRMA. In contrast, both 43DCRMA and 43CRMA exhibit higher non-recoverable strain, especially under 3.2 kPa, where cumulative deformation increases rapidly. These results further confirm the hybrid strategy effectively enhances deformation resistance at high temperatures.

### 3.5. Solubility Test and PE-IE Analysis

**Figure 7:**
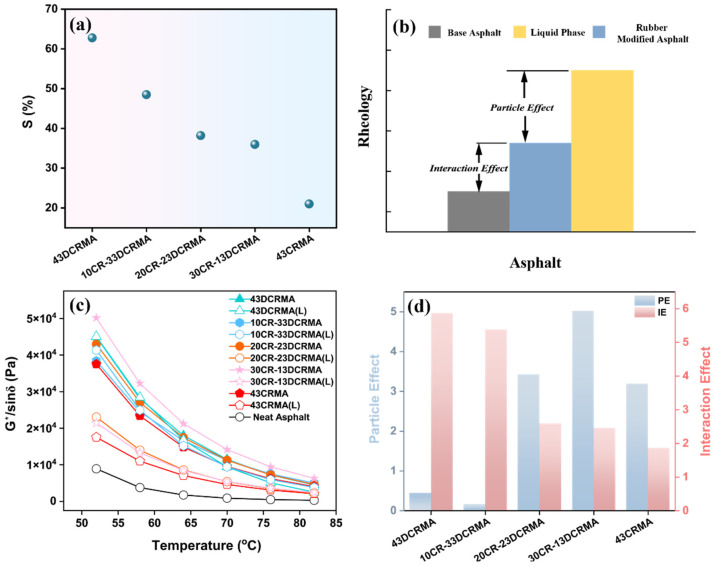
(**a**) Sol fraction variation in rubber-asphalt systems (room temperature); (**b**) diagram of particle effect (PE) and interaction effect (IE); (**c**) temperature sweeps of blended asphalt and liquid phases (L); (**d**) PE and IE values of rubber-modified asphalt with varied CR/DCR ratios.

We further explored the mechanism through which the CR-DCR hybrid strategy enabled improvement in asphalt performance. It is known that the macroscopic properties of rubber-modified asphalt are governed by microstructural changes, which result from the dissolution, swelling, and degradation of the incorporated rubber particles [36,38,39]. To probe the microstructural evolution of the rubber fillers within the asphalt, the sol content was quantified. Figure 7a presents the sol fraction of the extracted rubber particles; it is revealed that in 43DCRMA, rubber particles exhibit the highest solubility (60%), demonstrating their severe degradation, which may enhance their compatibility with asphalt. For modified asphalt with higher CR ratio, the solubility of the extracted rubber particles first decreases quickly and then reaches a plateau in 30CR-13DCRMA, indicating less degradation of the incorporated rubber particles. These less degraded rubber particles retain their elastic nature and can swell to reinforce the asphalt matrix. However, as the CR ratio exceeds 30 (43CRMA), the particles cannot reach a fully swollen state due to limited swelling space and are more prone to agglomeration (Figure S2).

The distinct swollen state of the rubber particles should contribute to their different reinforcement effects within the asphalt, which can be characterized by calculating the *PE* and *IE* values. A higher *PE* indicates a more pronounced reinforcement effect from the swollen rubber particles. A lower *IE* suggests poorer compatibility between rubber particles and the asphalt (Figure 7b) [53,54]. We further quantified the *PE* and *IE* values through rheological tests (Figure 7c). As shown in Figure 7d, 43DCRMA exhibits the lowest *PE* and a high *IE*, which can be attributed to severe degradation impairing elasticity, and its improved compatibility with the asphalt. As the CR ratio increases, the *PE* reaches a maximum in 30CR-13DCRMA, confirming the most effective reinforcement effect. As the CR ratio increases beyond 30, as in 43CRMA, both *PE* and *IE* decrease to their lowest values, signifying inadequate swelling and particle agglomeration weakening the reinforcement effect.

### 3.6. Gpc Test Analysis

**Figure 8:**
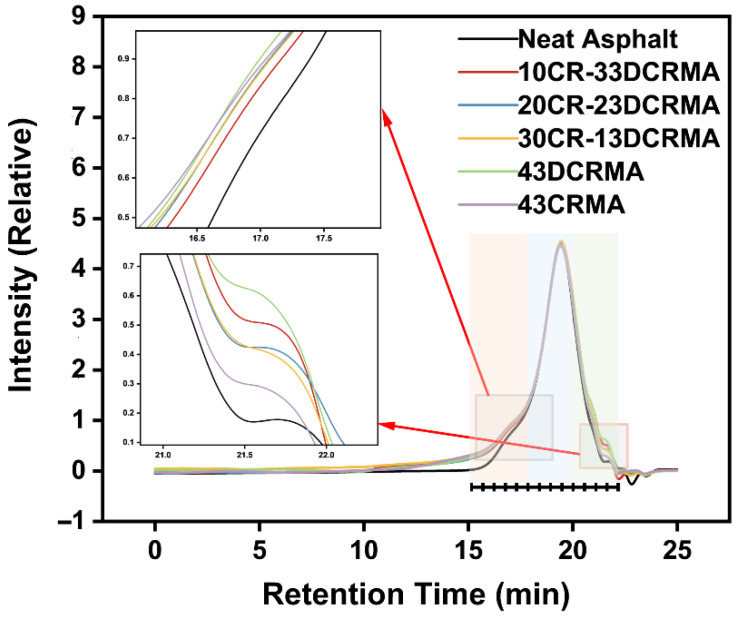
GPC of rubber-modified asphalt with varied formulations at room temperature.

A GPC test was further carried to reveal the structural evolution of rubber-modified asphalt from the perspective of molecular weight distribution. The GPC curves were partitioned into three parts (13 slices) based on retention time: the large molecular size (LMS) region (slices 1–5), medium molecular size (MMS) region (slices 6–9), and small molecular size (SMS) region (slices 10–13). A higher molecular weight corresponds to a shorter retention time. The fractional area under each curve corresponds to the relative abundance of molecules within each size range, with the LMS fraction representing macromolecular content and the SMS fraction reflecting small-molecule constituents. As shown in Figure 8, the largest peak area in SMS of 43DCRMA can be attributed to the severe degradation of DCR during processing (Figure S3), which led to the release of low molecular chains of rubber into the asphalt matrix [55]. This observation is consistent with the higher IE values for 43DCRMA. With increasing proportion of CR, the less degraded rubber particles resulted in smaller SMS areas [56]. In comparison, the LMS areas exhibit an opposite trend with increasing CR proportion, as more rubber particles participate in absorbing light components in the asphalt matrix to swell. It is well established that the LMS fraction in asphalt contributes more significantly to the enhancement of the physical properties of modified asphalt.

### 3.7. AFM Analysis

**Figure 9:**
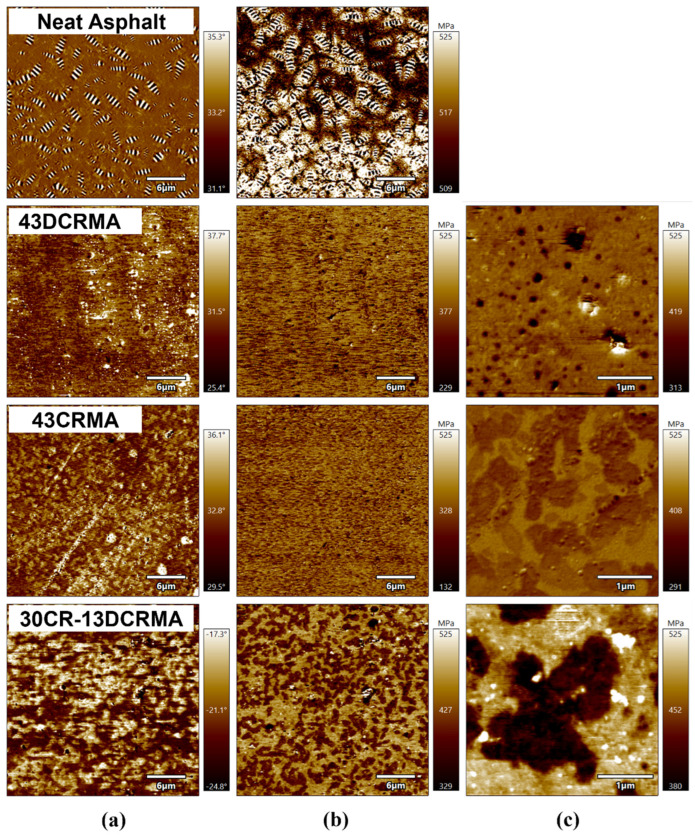
AFM topography images of crumb-rubber-modified asphalt with varied formulations at room temperature: (**a**) phase angle images, (**b**) modulus maps, (**c**) amplified modulus maps.

AFM was further employed to investigate the micromorphological evolution of the high-content rubber-modified asphalt with CR/DCR hybridization. The phase and modulus distribution images are presented in Figure 9. From Figure 9a, it can be observed that unmodified asphalt exhibits typical “bee structures”, which originate from the presence of stiff microcrystalline asphaltenes and paraffin within the asphalt matrix [57,58]. In comparison, those “bee structures” disappear in rubber-modified asphalt. This is because the incorporation of rubber particles promotes the absorption of light fractions within asphalt matrix, suppressing the wax-induced formation of “bee structures”.

Additionally, 43DCRMA shows a more homogeneous phase and lower modulus. This phenomenon is attributed to the severe degradation of DCR, which generates small molecules and improves the compatibility between rubber particles and the asphalt matrix, consistent with prior GPC and solubility analyses. In the case of 43CRMA, a uniform and slightly phase-separated structure is observed, resulting from the poor compatibility between rubber particles and the asphalt matrix. The high CR content requires substantial absorption of light fractions to achieve full swelling, which increases the relative concentration of continuously aggregating resins and asphaltenes, the AFM results for 10CR-33DCRMA and 20CR-23DCRMA confirm this trend (Figure S4). This is manifested in the bright high-modulus regions visible in modulus maps [59]. For 30CR-13DCRMA, however, the hybridization strategy leads to a distinct phase structure and modulus distribution within the asphalt matrix. The hybridization of CR and a small portion of DCR allows for improved absorption of light components and small molecules, facilitating the agglomeration of the resin and asphaltene components [60], as evidenced by the high-modulus regions in Figure 9c. This distinct microstructural evolution is likely responsible for the improved physical properties of modified asphalt.

### 3.8. Mechanism of the CR/DCR Hybridization’s Improved Performance

**Figure 10:**
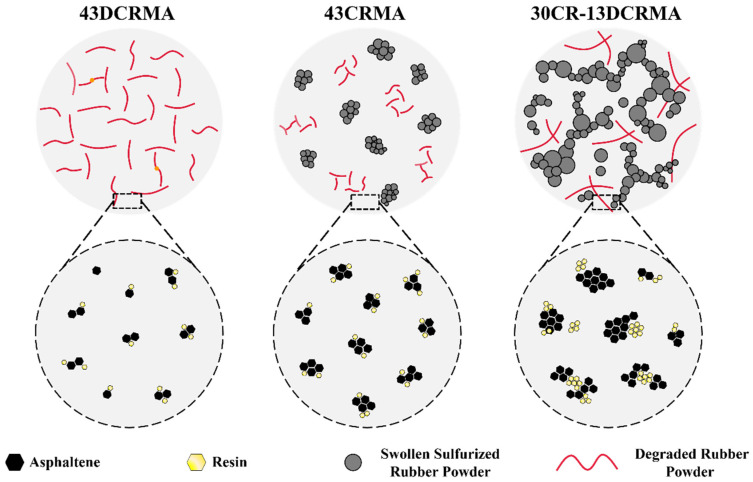
Schematic diagrams of modification mechanism of CR/DCR hybridization.

Building upon the aforementioned rheological, GPC, dissolution, and AFM characterizations, the CR/DCR-hybridization-enabled improvement in the performance of high-content rubber-modified asphalt can be depicted in Figure 10. In the case of 43DCRMA, the incorporated rubber fillers undergo severe degradation and produce more low-molecular-weight chains. Although these degraded chains exhibit improved compatibility and dissolve more easily into the asphalt matrix, they cannot produce enough LMS components. In stark contrast, due to their highly cross-linked structures, the rubber particles in 43CRMA cannot fully absorb those light components to reach their fully swollen state. As a result, rubber particles remain in a severely aggregated form and have limited influence on the microstructure of the asphalt matrix. Fortunately, the hybridization of CR and a small portion of DCR promotes more effective absorption of the light components, facilitating full swelling of the rubber particles and altering the microstructure of the asphalt matrix through the formation of resin- and asphaltene-enriched domains (30-13DCRMA). Consequently, the modified asphalt exhibits remarkably improved physical properties, even with a rubber content as high as 43 wt%.

## 4. Conclusions

This study proposes a CR/DCR hybridization strategy for fabrication of high-performance modified asphalt with a rubber content as high as 43 wt%. Comprehensive characterization revealed the following conclusions:

(1) Hybrid-rubber-modified asphalt demonstrated superior fundamental properties over single-type counterparts. The optimal performance was achieved in the 30CR-13DCRMA formulation, which exhibited a softening point of 78.4 °C, enhanced ductility of 15.33 cm, and a reduced softening point difference of only 0.15 °C.
(2) Compared with neat DCR- or CR-modified asphalt, 30CR-13DCRMA exhibited enhanced high-temperature rutting resistance and fatigue tolerance. Its non-recoverable creep compliance of under 0.1 kPa and 3.2 kPa decreased to 0.99 and 2.37 kPa^−1^, respectively, while maintaining excellent elastic recovery performance.
(3) Based on rheological analysis, solubility tests, GPC and AFM characterization, the underlying mechanism responsible for the improved physical properties of high-content rubber-modified asphalt can be explained as follows: The hybrid strategy enables maximized absorption capabilities of rubber particles to enable their full swelling, while altering the microstructure of the asphalt matrix with resin/asphaltene-enriched domains.

This study should inspire the design of high-performance asphalt with large proportions of waste rubber particles for sustainable pavement applications.

## Figures and Tables

**Table 1:** Physical properties of 90^#^ base asphalt.

Properties	Test Values
Penetration (25 °C, 0.1 mm)	73
Softening point (°C)	45.4
Ductility (5 °C, cm)	0
Brook Viscosity (180 °C, Pa·s)	0.12

**Table 2:** Relevant parameters of devulcanized crumb rubber (DCR) and neat crumb rubber (CR).

Components	DCR	CR
Rubber hydrocarbon (wt%)	55.51	57.13
Carbon-black (wt%)	27.18	27.37
Ash (wt%)	9.58	6.88
Oil (wt%)	7.68	8.6
Sol (%)	32	—

**Table 3:** Hybrid-rubber powder formulations.

Samples	CR Content (wt%)	DCR Content (wt%)	Total Content (wt%)
43 wt% DCR Modified Asphalt	0	43	43 wt%
10 wt% CR/33wt% DCR Modified Asphalt	10	33	
20 wt% CR/23wt% DCR Modified Asphalt	20	23	
30 wt% CR/13wt% DCR Modified Asphalt	30	13	
43 wt% CR Modified Asphalt	43	0	

Note: All five sample groups will be uniformly abbreviated in subsequent sections by removing unit designations and forming acronyms from initial capital letters, e.g., 43wt% DCR-modified asphalt represents 43DCRMA.

## Data Availability

The original contributions presented in this study are included in the article/Supplementary Material. Further inquiries can be directed to the corresponding authors.

## Supplementary Materials

The following supporting information can be downloaded at https://www.mdpi.com/article/10.3390/polym17222987/s1: Figure S1: Excessive crumb rubber content (45 wt%) impeded fabrication of modified asphalt; Figure S2: crumb SEM images of the 43CRMA and 43DCRMA; Figure S3: GPC of CR and DCR used in this work; Figure S4: AFM Topography Images of crumb rubber modified asphalt with varied formulations; Table S1: Influence of processing parameters on the fundamental physical properties of modified asphalt. References [14,61] are cited in the supplementary materials.
