# Supplementary material for: Hybrid Devulcanized/Vulcanized Crumb Rubber Strategy for High-Performance Asphalt with over 40% Recycled Tire Rubber Content
Source: Polymers (Basel). 2025 Nov 10;17(22):2987. doi: 10.3390/polym17222987 (PMC12656086; doi:10.3390/polym17222987)
Supplement: Supplementary file 1 [file polymers-17-02987-s001.zip › polymers-3964217-supplementary.pdf]

Supporting Information

for

Hybrid Devulcanized/vulcanized Crumb Rubber Strategy for High-Performance Asphalt with over 40% Recycled Tire Rubber Content

Zhengkun Wang[a], Ruihuan Wang[a], Heng Zhang[a]*, Bo Zhang[b], Yinghua Fan[c], Wenwen Yu[a], Qiang Zheng [a,d], Fengbo Zhu[a]*

[a] *College of Materials Science & Engineering, Taiyuan University of Technology, Taiyuan 030024, China*

[b] *Shanxi Transportation Technology Research and Development Co., Ltd., Taiyuan 030032, China*

[c] *The Key Laboratory of Road and Traffic Engineering, Ministry of Education, Tongji University, Shanghai, 201804, China*

[d] *Department of Polymer Science & Engineering, Zhejiang University, Hangzhou 310027, China*

Table S1 Influence of processing parameters on the fundamental physical properties of modified asphalt

Preparation Process	Softening point (°C)	Penetration (0.1 mm)	Ductility (cm)	Elastic recovery(%)	Viscosity (Pa·s)
200°C<br>Stir for 60 min<br>Shear for 50 min	67.6	61.2	9.09	79.65	2.83
200°C<br>Stir for 60 min<br>Shear for 30 min	68.9	58.3	7.92	80.30	3.06
190°C<br>Stir for 60 min<br>Shear for 50 min	73.2	55.1	10.8	80.17	3.43
190°C<br>Stir for 60 min<br>Shear for 30 min	76.1	51.7	15.41	82.83	4.02

Based on literature review and problems encountered during preliminary preparation, the pre-experimental conditions were established[1], It was observed that at 180 °C, the modified asphalt required a long time to reach uniform mixing, and the product exhibited excessive viscosity. To balance viscosity and achieve better overall performance, the preparation temperature was set between 190 °C and 195 °C, with a shearing time of 30–50 min (typically 40 min).

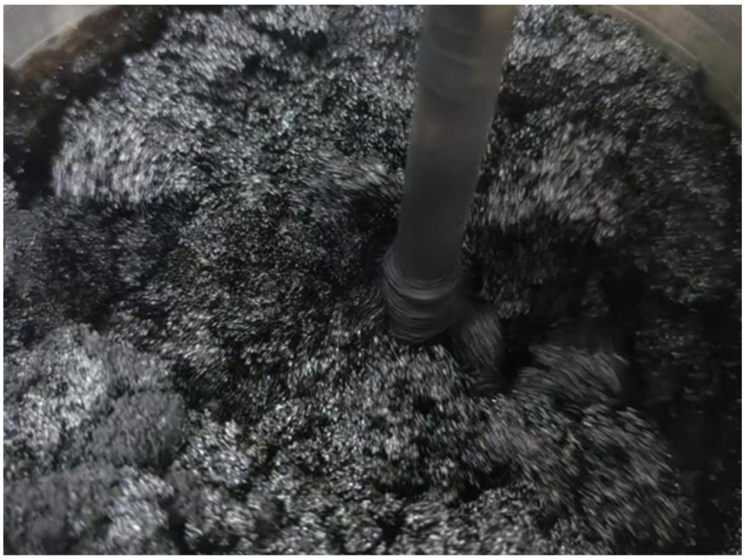

Figure S1. Excessive crumb rubber content (45 wt%) impeded fabrication of modified asphalt.

Within the experimental parameters of this study, we aimed to incorporate a greater proportion of rubber powder into the asphalt matrix. The findings revealed that when the rubber powder content exceeded 43%, it became challenging to maintain stable experimental conditions, thus hindering the normal progression of our investigations.

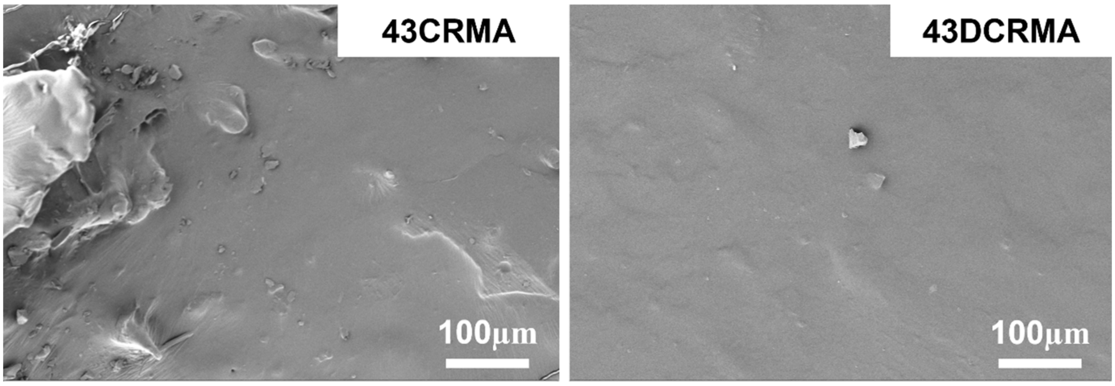

Figure S2. SEM images of the 43CRMA and 43DCRMA

The fracture surface of 43DCRMA is smooth and even, which can be attributed to the homogeneous dispersion of the devulcanized crumb rubber (DCR) within the asphalt matrix. In contrast, the untreated crumb rubber (CR) exhibits poor compatibility with asphalt, resulting in a rough fracture surface with evident rubber agglomeration in the 43CRMA sample.

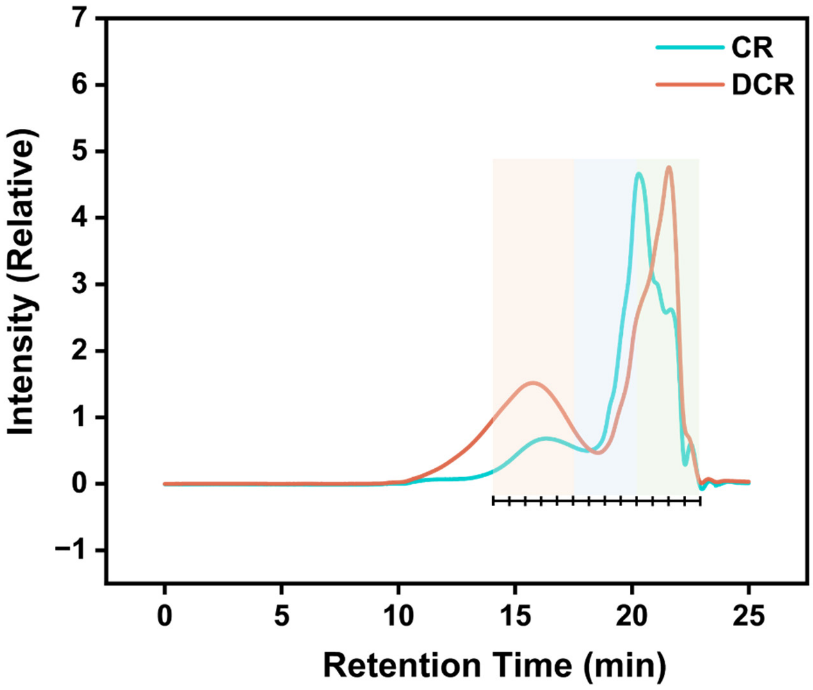

Figure S3. GPC of CR and DCR used in this work.

Figure S3 shows increased LMS region in DCR GPC profile due to cross-link disruption during devulcanization, generating linear macromolecules. In contrast, intact cross-linked network of CR renders it THF-insoluble, removing high-MW components during filtration. Consequently, CR GPC signal originates solely from low-MW soluble fractions, showing weak LMS but strong SMS response[2]. Thus, regarding the results presented in Figure 8 of the main text, We deduce the large linear chains of DCR directly increase macromolecular content in modified asphalt. Conversely, CR absorbs the light components (small molecules) of asphalt to swell, causing localized enrichment of large molecules (e.g., asphaltenes).

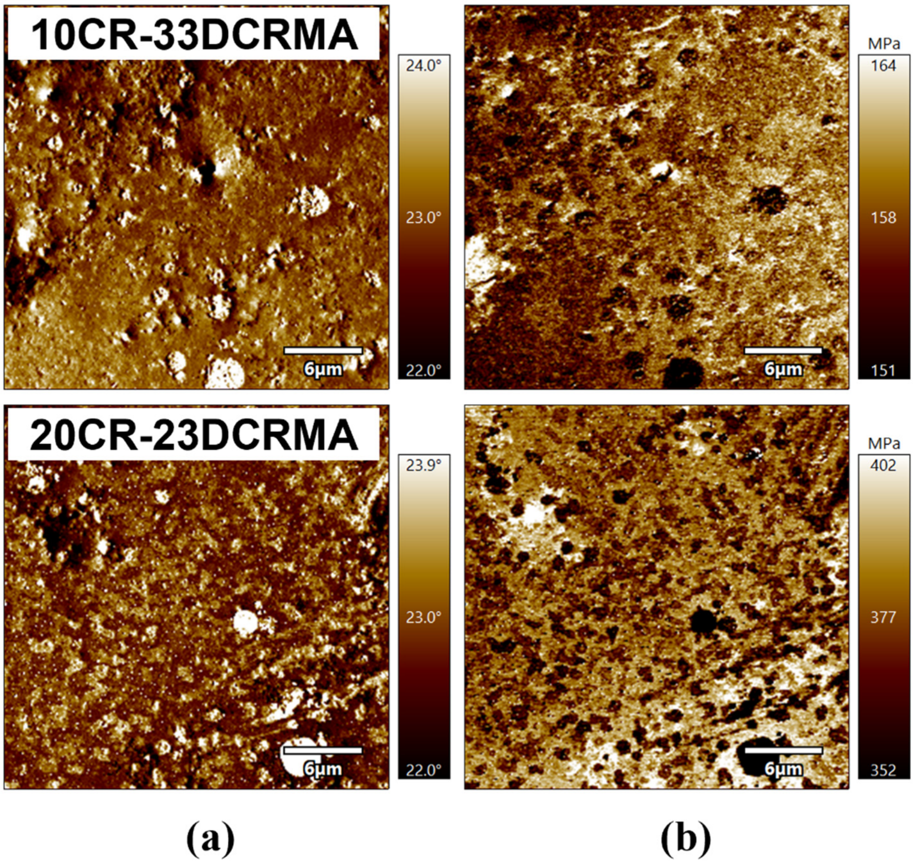

Figure S4. AFM Topography Images of crumb rubber modified asphalt with varied formulations: (a) phase angle images (b) modulus maps

As outlined in the main text (**Figure 9**), crosslinked crumb rubber (CR) exhibits a propensity to absorb lighter fractions from the asphalt during the swelling process. When the CR content is relatively low (10CR-33DCRMA), the modified asphalt displays a soft and ductile behavior, with its modulus values closely aligning with those of the 43DCRMA. However, as the quantity of CR swelling increases, a notable enhancement in the modulus of the modified asphalt is observed (20CR-23DCRMA), resulting in a progressively interconnected soft-hard phase structure. This change is attributed to the increased absorption of low-molecular-weight, mobile components within the asphalt by the CR, which facilitates the aggregation of higher molecular weight entities.

References

[1] Duan, K.X.; Wang, C.H.; Liu, J.K.; Song, L.; Chen, Q.; Chen, Y.Z. Research progress and performance evaluation of crumb-rubber-modified asphalts and their mixtures. *Constr. Build. Mater.* **2022**, *361*, 129687.

[2] Wang, R.; Wang, Z.; Cui, C.; Yu, W.; Zhu, F.; He, H.; Du, H.; Liao, L.; Duan, D.; Zhang, B.; Fan, Y. Secondary relaxation-driven self-healing optimization in desulfurized crumb rubber-modified asphalt. *Constr. Build. Mater.* **2025**, *489*, 142370.
